# The impact of complete surgical resection of spinal metastases on the survival of patients with thyroid cancer

**DOI:** 10.1002/cam4.823

**Published:** 2016-07-19

**Authors:** Satoshi Kato, Hideki Murakami, Satoru Demura, Yoshiyasu Fujimaki, Katsuhito Yoshioka, Noriaki Yokogawa, Hiroyuki Tsuchiya

**Affiliations:** ^1^ Department of Orthopaedic SurgeryKanazawa University School of MedicineKanazawaJapan

**Keywords:** Metastasectomy, spinal metastases, spondylectomy, surgical resection, survival, thyroid carcinoma

## Abstract

Spinal metastases (SMs) from thyroid cancers significantly reduce the quality of life by causing pain and neurological deficits and increase mortality. Complete surgical resection of isolated thyroid SMs is a promising treatment option; however, the postoperative outcome is unknown. This study aimed to compare the postoperative courses of patients undergoing complete resection of thyroid SMs with those of patients undergoing incomplete resection, with a minimum 4‐year follow‐up. We performed a retrospective analysis of 32 patients who underwent tumor excision surgery for thyroid SMs at our medical center during a 28‐year period. Twenty patients underwent complete excision, and 12 underwent incomplete excision. Survival was defined as the time from the first spinal surgery to death or last follow‐up. Kaplan–Meier analysis with the long‐rank test was used to compare the overall survival rates between the groups. For all patients, the overall 5‐ and 10‐year survival rates were 71% and 31%, respectively. The median overall survival time was 6.4 years. The patients undergoing complete excision survived longer than those undergoing incomplete excision (5‐year survival: 84% vs. 50%; 10‐year survival: 52% vs. 8%; *P *< 0.01). Only one patient undergoing complete excision experienced local tumor recurrence in the operated spine, whereas all long‐term survivors (>18 months after surgery) in the incomplete excision group experienced local tumor recurrence and a consequent deterioration in performance status. Complete surgical resection of thyroid SMs, if achievable, has the potential not only to maintain performance status, but also to prolong survival.

## Introduction

Thyroid carcinoma (TC) is generally not aggressive and is associated with a relatively favorable long‐term survival 1. However, the presence of distant metastases reduces the 10‐year overall survival rate to between 13% and 42% 1, 2, 3, and distant metastases are the most frequent cause of TC‐related death 2, 4.

Bone metastases (BMs) from differentiated TCs appear in 2–13% of patients 1, 3. Spinal metastases (SMs) are the most common type of thyroid BMs, accounting for approximately 50% of all BMs 5, 6, 7. Although thyroid SMs have the most favorable prognosis of all tumors metastasizing to the spine 2, 8, 9, they often cause intractable pain, neurological deficits, and paraplegia, thus substantially reducing the quality of life, and increase mortality 2, 6, 7. Moreover, most thyroid BMs with osteolytic lesions are destructive and more resistant to systemic therapy and radiation than other organ metastases. SMs in particular result in pathologic fractures and spinal cord compression that substantially impair the performance status of patients.

A significant proportion of patients with thyroid SMs have a solitary spinal lesion without nonspinal BMs nor other organ metastases and are eligible for aggressive surgical treatment, including metastasectomy, which is intended to improve their quality of life and prolong survival 10. However, despite advancements in surgical techniques and materials in recent years, complete excision of tumor‐affected vertebrae (spondylectomy) and en bloc excision of tumors in the spine are still more technically demanding than are resections of tumors in other parts of the body. In fact, most spine surgeons only perform palliative spinal cord decompression plus instrumented stabilization.

**Figure 1:**
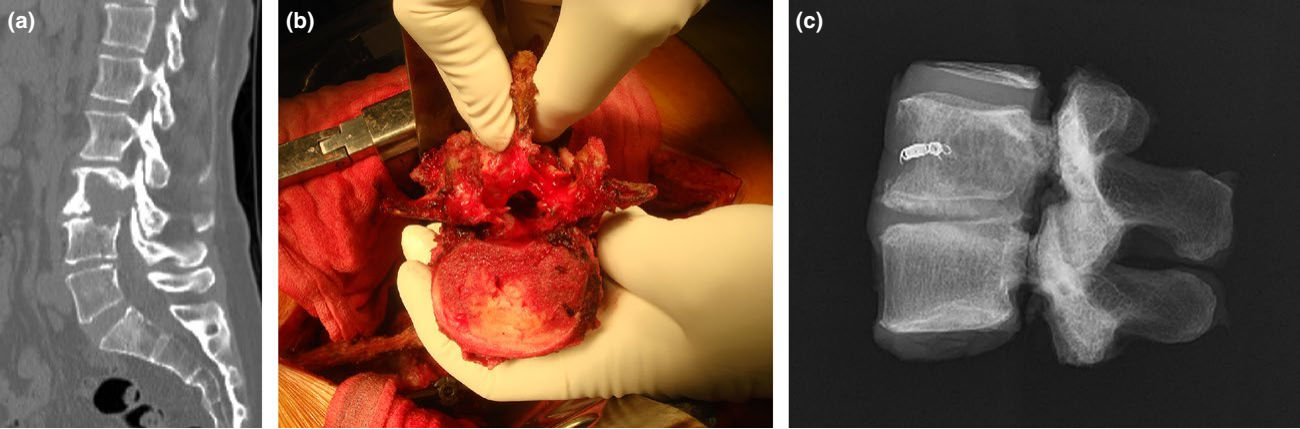
Representative case of total en bloc spondylectomy. (A) Sagittal computed tomography showing a tumor involving L3 and L4. (B) Intraoperative photograph of the resected specimen. (C) Radiograph of the specimen showing the tumor resected en bloc.

Spondylectomy as a curative surgery for spinal tumors was first reported by Stener 11. In the early 1990s, Tomita et al. from our institute developed and standardized total en bloc spondylectomy (TES), which allows complete resection of tumor‐affected vertebrae 12. TES consists of dorsal en bloc resection after transpedicular osteotomy and subsequent ventral vertebrectomy (Fig. 1). Improvements in surgical techniques and standardization of preoperative arterial embolization have provided favorable results with low morbidity 13, and TES has been performed in selected patients with a solitary SM 14, 15, 16. This study aimed to compare the ≥4‐year postoperative courses of patients who underwent complete resection of thyroid SMs (mainly via TES) and patients who underwent incomplete resection, and to evaluate the impact on survival of complete surgical resection of thyroid SMs.

## Materials and Methods

### Patients

From 1984 and 2011, 352 patients with SMs were surgically treated at our institution. After approval by the institutional review board, a retrospective review identified 32 patients with TCs who underwent complete excision of SMs (complete excision group, 20 patients) or incomplete excision of SMs (incomplete excision group, 12 patients). In the complete excision group, TES was performed in 19 patients, and total excision of the posterior spinal elements in one patient whose isolated spinal metastasis involved only the posterior elements of the first and second thoracic vertebrae. The characteristics of the patients in the two groups are presented in Table 1. At the time of spinal surgery, nine patients (28%) had vital organ metastases, all of which were located in the lung. No patient had metastases in the liver or brain. Only four patients (12.5%, one in the complete excision group and three in the incomplete excision group) had multiple lesions in the spine.

**Table 1:** Patient characteristics of the two groups

Characteristic	Complete excision	Incomplete excision	*P* value
No. of patients	20	12	
Men/women, No.	2/18	5/7	
PTC/FTC/MTC, No.	6/13/1	4/8/0	
Age (years), mean ± SD	59.4 ± 10.3	62.5 ± 7.9	0.37
Tokuhashi score	12.9 ± 1.7	12.1 ± 1.4	0.17
Tomita score	2.9 ± 1.1	2.8 ± 0.7	0.85
Vital organ metastases, No. (%)	6 (30%)	3 (25%)	0.55
Multiple metastases, No. (%)	12 (60%)	8 (66%)	1.00
Multiple SMs, No. (%)	1 (5%)	3 (25%)	0.14
Synchronous SMs, No. (%)	5 (25%)	4 (33%)	0.70
Cervical or L5 lesion, No. (%)	1 (5%)	6 (50%)	0.02
Revision surgery (a), No. (%)	1 (5%)	3 (25%)	0.14
Radioiodine therapy, No. (%)	13 (65%)	7 (58%)	0.50
External radiotherapy, No. (%)	5 (25%)	4 (33%)	0.45
Bisphosphonate or denosumab administration, No. (%)	9 (45%)	5 (42%)	0.85

PTC, papillary thyroid carcinoma; FTC, follicular thyroid carcinoma; MTC, medullary thyroid carcinoma; SD, standard deviation; SM, spinal metastases; L5, the fifth lumbar vertebra.

a In the 4 patients, we performed revision surgery for symptomatic tumor recurrence after surgery at another hospital.

TES involving the cervical spine or the fifth lumbar spine is technically demanding and sometime impossible owing to the anatomical architecture. Compared with other spinal levels, TES at these spinal levels inflicts greater surgical stress on the patients and may lead to higher morbidity, with increased operative time and blood loss. Moreover, TES as a revision surgery for tumor recurrence after the initial surgery is sometimes not feasible. For these reasons, a greater proportion of patients in the incomplete excision group had metastases in the cervical spine or the fifth lumbar spine, and had tumor recurrence after surgery at another hospital (Table 1). Incomplete excision surgery was performed for all four patients who underwent surgery in the 1980s, as procedures for the complete excision of diseased vertebrae, such as TES, were not feasible at that time.

### Surgical procedure

TES involved en bloc excision of spinal tumors along with the surrounding musculoligamentous supportive tissue that served as a barrier to tumor progression. The TES technique consists of two steps to salvage the spinal cord: en bloc excision of the dorsal part of the vertebra(e) after transpedicular osteotomy and subsequent en bloc excision of the ventral part. In some cases, a small part (the pedicles of the vertebral arch in most cases) is intentionally made intralesional, which is necessary to salvage the spinal cord. The surgical approach is decided on the basis of the degree of tumor development or the affected spinal levels. The technique aspects of TES have been described in detail elsewhere 13.

Incomplete excision included subtotal piecemeal excision or eggshell curettage of the spinal tumor. Most of the tumor was removed in an intralesional fashion, whereas the shell of the vertebral column and the surrounding supportive tissue were preserved. Cases of palliative spinal cord decompression by laminectomy only were excluded from this study.

### Data analysis

We evaluated the survival, performance of activities of daily living (ADL), and ambulatory status during the postoperative courses of the 32 patients in our study. Survival was examined from the time from the first SM surgery to death or the last follow‐up. The performance of ADL was evaluated using the Karnofsky Performance Status (KPS) scoring system. The Kaplan–Meier analysis method was used to estimate postoperative survival, and survival curves were compared using the log‐rank test.

To identify factors associated with survival, we analyzed the following clinical factors: complete resection of SMs, papillary histology, lung metastases, multiple metastases and multiple SMs at spine surgery, synchronous SMs with primary lesions, history of radioiodine therapy, external radiotherapy, and bisphosphonate or denosumab administration. Multivariate analysis using the Cox proportional hazards model was performed to assess the relationship between survival time and several variables simultaneously. Variables in a univariate analysis with a *P* ≤ 0.20 were entered into the multivariate analysis. Univariate analysis to compare patient characteristics between the complete and incomplete excision groups was performed using Student's *t*‐test or Fisher's exact test (Table 1). The data were analyzed using SPSS software version 16.0 for Windows (SPSS Inc., Chicago, Illinois), and the significance level was set at *P* < 0.05.

## Results

### Survival

**Figure 2:**
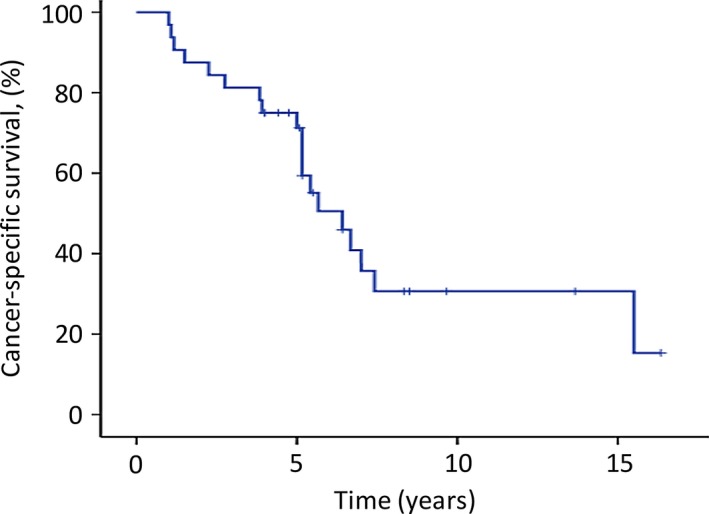
The overall survival of the 32 patients undergoing surgical resection of spinal metastases from thyroid cancer. The 5‐ and 10‐year cancer‐specific survival rates were 71% and 31%, respectively. The tick marks indicate the last dates of follow‐up.

**Figure 3:**
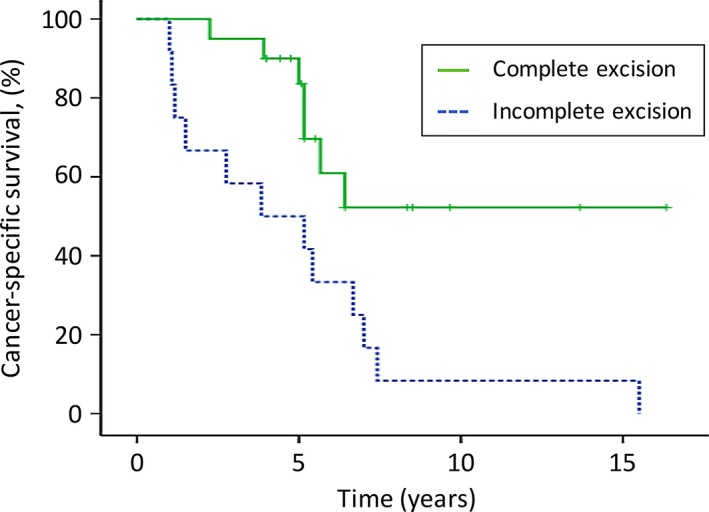
Comparison of the overall survival of the complete excision group with that of the incomplete excision groups. The patients in the complete excision group survived longer than those in the incomplete excision group (5‐year survival: 84% vs. 50%; 10‐year survival: 52% vs. 8%; *P* < 0.01). The tick marks indicate the last dates of follow‐up.

**Figure 4:**
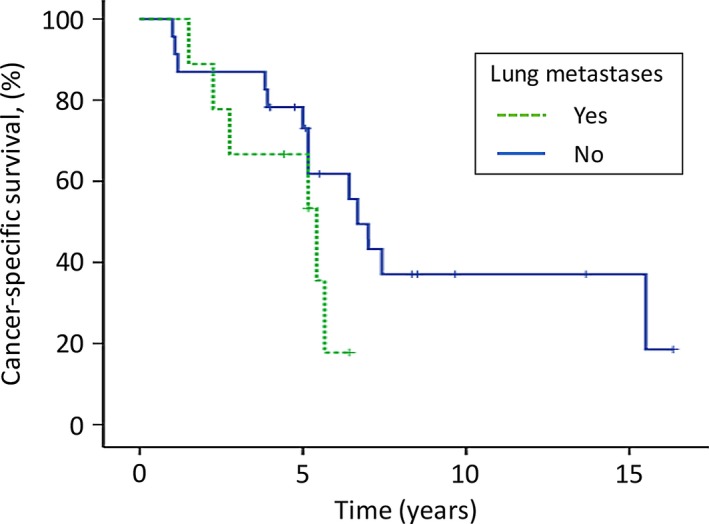
Comparison of the overall survival of patients with lung metastases with that of patients without lung metastases. No significant difference was observed between the two groups. The tick marks indicate the last dates of follow‐up.

At the last follow‐up (≥4 years after the surgery in our hospital), 13 (65%) of the 20 patients in the complete excision group were still alive (survival time after surgery: 4.0–16.3 years), whereas all 12 patients in the incomplete excision group had died of the disease. The median overall survival time of the 12 patients in the incomplete excision group was 3.8 years (range: 1.0–15.5 years). For all 32 patients, the overall 5‐ and 10‐year survival rates after the initial SM surgery were 71% and 31%, respectively (Fig. 2). The median overall survival time was 6.4 years. The patients in the complete excision group survived significantly longer than those in the incomplete excision group (5‐year survival: 84% vs. 50%; 10‐year survival: 52% vs. 8%; *P *= 0.006; Fig. 3 and Table 2). The median overall survival time of patients with lung metastases was 5.4 years (5‐year survival rate, 67%), which was shorter than that of patients who had no lung metastases (6.7 years; 5‐year survival rate, 73%). However, no significant difference was observed between the two groups (*P* = 0.171; Fig. 4 and Table 2).

**Table 2:** Univariate analysis to identify factors associated with survival

Factor	*n* (%)	5 (& 10)‐year survival rate, %	*P* value
Surgery of spinal metastaes			
Complete resection	20 (62.5)	83.6, (52.2)	0.006
Incomplete resection	12 (37.5)	50.0, (8.3)	
Histology			
Papillary	10 (31.2)	50.0, (18.8)	0.047
Nonpapillary	22 (68.8)	81.0, (35.5)	
Lung metastases at spine surgery			
Yes	9 (28.1)	66.7, (17.8)	0.171
No	23 (71.9)	73.0, (37.1)	
Extent of metastases at spine surgery			
Single in the spine	12 (37.5)	83.3, (47.6)	0.070
Multiple (spine or other sites)	20 (62.5)	64.2, (20.0)	
Extent of spinal metastases at spine surgery			
Single	28 (87.5)	75.0, (37.2)	0.104
Multiple	4 (12.5)	50.0, (25.0)	
Type of spinal metastases			
Synchronous	9 (28.1)	77.8, (16.7)	0.380
Metachronous	23 (71.9)	68.2, (38.4)	
Radioiodine therapy			
Yes	20 (62.5)	75.0, (20.5)	0.553
No	12 (37.5)	64.3, (42.9)	
External radiotherapy			
Yes	9 (28.1)	77.8, (46.7)	0.797
No	23 (71.9)	69.0, (25.9)	
Bisphosphonate or denosumab administration			
Yes	14 (43.7)	63.5, (39.7)	0.857
No	18 (56.3)	77.8, (24.7)	

In the univariate analysis, complete excision (*P* = 0.006) and papillary histology (*P* = 0.047) significantly influenced postoperative overall survival (Table 2). The presence of multiple metastases (*P* = 0.070), multiple spinal metastases (*P* = 0.104), and lung metastases (*P* = 0.171) at spine surgery had near‐significant associations with short‐term survival (Table 2). In the multivariate Cox analysis, only complete excision was an independent factor for survival after spinal tumor excision surgery (relative death risk, 0.258 [95% confidence interval: 0.100–0.667], *P* = 0.005).

### Local recurrence and performance of ADL

Nine (75%) of the 12 patients in the incomplete excision group experienced symptomatic local recurrence in the operated spine, at a mean period of 4.1 years after the initial surgery. The other three patients died within 18 months after the surgery. Eight (89%) of the nine patients with symptomatic local recurrence in the incomplete excision group underwent additional tumor excision surgeries (total, 13 surgeries; mean, 1.6 times). Owing to symptomatic local recurrence and/or repeated tumor excision surgery, the performance of activities of daily living (ADL) in these eight patients deteriorated, and all died of the disease at a mean period of 16 months (range: 1–26 months) after the last surgery. The remaining patient with symptomatic local recurrence in the partial excision group had multiple undifferentiated metastases and thus did not undergo additional tumor excision surgery; he died of the disease 2 months after the recurrence.

In contrast, only one of the 20 patients in the complete excision group experienced symptomatic local recurrence in the operated spine, 33 months after surgery. He underwent additional tumor excision surgery and died of the disease 29 months after the second surgery. In the 13 patients in the complete excision group who were still alive at the latest follow‐up, four had no evidence of disease, and the other nine were alive with disease. Twelve of the 13 patients were ambulatory and maintained their ADL without assistance (KPS score ≥70%) ≥4 years after surgery (range, 48–196 months).

### Case presentation

Figure 1 shows a representative case. The patient was a 52‐year‐old woman with a solitary metastasis of the lumbar spine. She underwent total thyroidectomy for a papillary carcinoma 19 months before admission to our hospital. On admission, she had significant lower back pain, which impaired her performance of ADL (KPS score: 70%). However, she did not have any neurologic deficits. Computed tomography and magnetic resonance imaging showed an osteolytic bone metastasis involving the 3rd and 4th lumbar vertebrae (Fig. 1). She underwent TES involving a posterior‐anterior combined approach (Fig. 1). One hundred months after the surgery, her performance of ADL was nearly normal (KPS score: 90%). She underwent radioiodine treatment for multiple lung metastases. Postoperative radiological images of the lumbar spine demonstrated that the reconstructed lumbar spine was well maintained, and no local tumor recurrence in the operated spine or newly developed BMs was observed.

## Discussion

Osteolytic and radiation‐resistant thyroid SMs cause neurological deficits, intractable pain, and consequent deterioration of the performance status of patients. A retrospective analysis of 245 cases of thyroid BMs demonstrated that 78% of the patients either presented with or developed at least one skeletal‐related event (SRE) after the diagnosis of BMs, and that the median time from the identification of BMs to the first SRE was 5 months 7. However, another retrospective analysis of 202 cases of SMs from TCs found that 54% of patients with single‐site SMs at the time of presentation had no other distant metastases 10. These distinguishing features and the relatively favorable prognosis of patients with metastases form the basis for aggressive surgical treatment of thyroid SMs. The American Thyroid Association guidelines state that complete removal of BMs can prolong survival and is particularly appropriate for younger patients 17, 18. Moreover, reduced performance of ADL and the neurological deficits caused by SMs make it difficult for patients to undergo radioiodine therapy, which is the mainstay of treatment for metastases from TC. Thus, complete surgical resection of SM, if achievable, should be considered. However, this aggressive surgery should be applied for patients with metastases from TC due to its distinguishing features and favorable prognosis. Treatment strategy for thyroid SMs is different from that for SMs from other malignancies.

Several studies have examined the postoperative survival of patients with thyroid SM 15, 19, 20, 21. The novel features of this study are its comparison of the long‐term survival of patients with thyroid SMs undergoing curative resection versus incomplete resection, and its evaluation of the importance of curative surgical resection of the metastases. In a previous retrospective analysis of 146 cases of thyroid BMs, the overall 5‐ and 10‐year survival rates from the time of diagnosis of the BMs (metastatic survival) were 25% and 13%, respectively;^6^ in a study of 109 cases, they were 41% and 15%, respectively 22. In this study, the overall 5‐ and 10‐year survival rates from the initial SM surgery were 71% and 31%, respectively, for all patients. These favorable results most likely reflect the aggressive surgical resection of the SMs. Accordingly, the patients in the complete excision group survived significantly longer than did those in the incomplete excision group (5‐year survival: 84% vs. 50%; 10‐year survival: 52% vs. 8%). In addition, all nine patients in the incomplete excision group who survived for >18 months after the spinal surgery experienced symptomatic local recurrence in the operated spine and deteriorated performance of ADL, indicating that long‐term survivors with thyroid SMs have significant symptoms when the resection is incomplete. Therefore, complete surgical resection of SMs is a valid treatment options that can also prolong survival.

Finally, the presence of lung metastases was not associated with survival in this study (*P* = 0.17). Lung metastases respond to radioiodine treatment better than other organ metastases 23, 24, and, in selected patients, are treatable via pulmonary resection with low morbidity 25, 26. In fact, favorable survival of patients with lung metastases has been presented compared with those of patients with other organ metastases 27, 28, 29. The results of our study suggest that complete surgical resection of thyroid SMs is considerable even for patients with coexistence of lung metastases.

This study has limitations, including the small cohort size and the retrospective nature of the analysis, which potentially could have introduced bias. Furthermore, we could not obtain circumstantial information about the primary TCs (TMN staging and surgery of primary lesions), the systemic therapy for metastases (indication and duration), or the radioiodine sensitivity of metastases because in most patients, primary TCs treatments and nonsurgical treatments of metastases were conducted at other hospitals (the patients were admitted to our hospital for SM surgery only). Thus, it is possible that a selection bias exists owing to the relatively stable disease condition of the patients, which allowed their consideration for SM surgery. Complete excision of spinal tumors including TES is an extraordinary and technically demanding surgery for general spine surgeons. Our experiences cannot be generalized to all surgical centers. We recommend that this surgery should be performed in spine tumor centers by well‐experienced surgeons. However, despite these limitations, this study clearly shows that complete surgical resection of SMs should be considered for patients with surgically curative SMs. In addition, for selected patients, it can help maintain performance status in the long‐term and potentially prolong survival.

## Conclusion

Our results showed that patients in the complete excision group survived significantly longer than those in the incomplete excision group, and that all long‐term survivors in the incomplete excision group experienced tumor recurrence and a consequent deterioration in performance status. Hence, complete surgical resection of SMs, if achievable, has the potential not only to maintain performance status, but also to prolong survival.

## Conflict of Interest

The authors have no conflicts of interest.
